# Neonatal administration of a subanaesthetic dose of JM-1232(−) in mice results in no behavioural deficits in adulthood

**DOI:** 10.1038/s41598-021-92344-3

**Published:** 2021-06-18

**Authors:** Koji Iwanaga, Yasushi Satoh, Ryosuke Akai, Toshiaki Ishizuka, Tomiei Kazama, Takehiko Ikeda

**Affiliations:** 1 Department of Anesthesiology, National Defense Medical College, Tokorozawa, Saitama Japan; 2 Department of Biochemistry, National Defense Medical College, Tokorozawa, Saitama Japan; 3 Department of Pharmacology, National Defense Medical College, Tokorozawa, Saitama Japan; 4 Department of Anesthesia, Toyooka-Daiichi Hospital, Iruma, Saitama Japan

**Keywords:** Toxicology, Neurochemistry, Fear conditioning, Short-term memory, Spatial memory, Working memory, Social behaviour

## Abstract

In animal models, neonatal exposure of general anaesthetics significantly increases apoptosis in the brain, resulting in persistent behavioural deficits later in adulthood. Consequently, there is growing concern about the use of general anaesthetics in obstetric and paediatric practice. JM-1232(−) has been developed as a novel intravenous anaesthetic, but the effects of JM-1232(−) on the developing brain are not understood. Here we show that neonatal administration of JM-1232(−) does not lead to detectable behavioural deficits in adulthood, contrarily to other widely-used intravenous anaesthetics. At postnatal day 6 (P6), mice were injected intraperitoneally with a sedative-equivalent dose of JM-1232(−), propofol, or midazolam. Western blot analysis of forebrain extracts using cleaved poly-(adenosine diphosphate-ribose) polymerase antibody showed that JM-1232(−) is accompanied by slight but measurable apoptosis 6 h after administration, but it was relatively small compared to those of propofol and midazolam. Behavioural studies were performed in adulthood, long after the neonatal anaesthesia, to evaluate the long-term effects on cognitive, social, and affective functions. P6 administration to JM-1232(−) was not accompanied by detectable long-term behavioural deficits in adulthood. However, animals receiving propofol or midazolam had impaired social and/or cognitive functions. These data suggest that JM-1232(−) has prospects for use in obstetric and paediatric practice.

## Introduction

Accumulating evidence indicates that exposure to general anaesthetics at clinically relevant concentrations induces a widespread increase in neuronal apoptosis in the developing brain of a variety of animals, from rodents to rhesus monkeys^1–11^. These are not short-term effects. Long after anaesthetic exposure in infancy, behavioural deficits can be manifested in adulthood^1–5^, even though a significant increase in neuronal apoptosis is no longer evident^12^. Consequently, there is growing concern about the use of general anaesthetics in obstetric and paediatric practice.

JM-1232(−) ((−)-3-[2-(4-methyl-1-piperazinyl)-2-oxoethyl]-2-phenyl-3,5,6,7-tetrahydrocyclopenta[f]isoindole-1(2H)-one) has been developed as a novel intravenous anaesthetic; it has increased sedative potency, therapeutic index, and water solubility^13,14^. JM-1232(−) has a therapeutic index of > 38.5, which indicates a safety margin that is greater than propofol, midazolam, and thiopental^13,15,16^. JM-1232(−) also has a shorter elimination half-life than midazolam^17^. These characteristics make JM-1232(−) attractive for clinical use. However, the effects of JM-1232(−) on the developing brain are not understood.

Propofol and midazolam are intravenous anaesthetics which are of special interest because they are used widely in clinical practice^18,19^. Similar to many other intravenous anaesthetics, propofol and midazolam are thought to act at least in part by binding to GABA type A (GABA_A_) receptors^20^. However, the mechanisms of action are different^16^. Propofol binds multiple sites of GABA_A_ receptor^21^. When coapplied with GABA or other agonists, propofol potentiates the response of GABA_A_ receptor to the transmitter, slowing the channel-closing time^22^. In the absence of GABA, propofol also activate the GABA_A_ receptor directly, causing channel opening^23^. On the other hand, midazolam binds to the benzodiazepine site of GABA_A_ receptor and increases the frequency of channel opening in response to GABA^24^. JM-1232(−) binds to the benzodiazepine site of GABA_A_ receptors as does midazolam^16^, but it does not have a benzodiazepine structure.

Previous studies have shown that subanaesthetic doses of propofol and midazolam induce apoptosis in infant mouse brain^7,25^. In the present study, we sought to determine the short- and long-term effects of JM-1232(−) on the developing mouse brain and compare those effects with those of propofol and midazolam.

## Results

### Sedative effects of JM-1232(−), midazolam, and propofol in neonates

**Figure 1:**
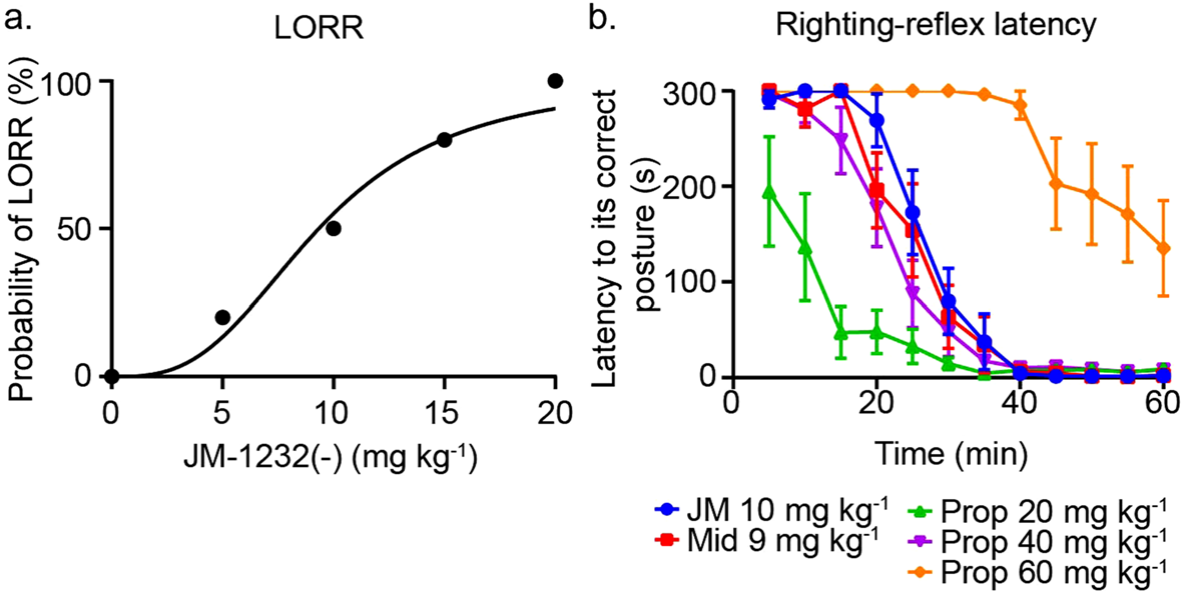
Sedative and analgesic effects of JM-1232(−) on P6 mouse pups and comparison with midazolam and propofol. (**a**) Loss of righting reflex (LORR) after a single intraperitoneal injection of JM-1232(−) at the indicated doses. The probability of LORR caused by a single intraperitoneal injection of JM-1232(−). LORR was evaluated 20 min after the injection. The following doses of JM-1232(−) (JM) were tested: 0 mg kg^−1^ (n = 5); 5 mg kg^−1^ (n = 5); 10 mg kg^−1^ (n = 6); 15 mg kg^−1^ (n = 5); 20 mg kg^−1^ (n = 5). (**b**) The depth of sedation elicited by the indicated anaesthetics was evaluated using the righting-reflex latency test. After a mouse pup was placed on its back, the length of time (latency) needed for it to correct its posture was measured every 5 min. The following anaesthetics and doses were tested: JM-1232(−) (JM); 10 mg kg^−1^ (n = 10), midazolam (Mid); 9 mg kg^−1^ (n = 10), propofol (Prop); 20 mg kg^−1^ (n = 6), 40 mg kg^−1^ (n = 11), 60 mg kg^−1^ (n = 8). Error bars are SEM.

To estimate an ED_50_ value of JM-1232(−) in mice at postnatal day 6 (P6), we evaluated the dose-dependent sedative effect using the loss of righting reflex (LORR) test. Mice were given a single injection of JM-1232(−) at a clinically relevant dose (n = 5 or 6 mice for each dose). JM-1232(−) had an ED_50_ of 9.3 mg kg^−1^ (95% confidence interval (CI) 5.7–12.7 mg kg^−1^) 20 min following injection (Fig. 1a). Thus, we roughly estimated that 10 mg kg^−1^ was within the subanaesthetic range for JM-1232(−) in P6 mice.

To determine the sedative-equivalent dose of JM-1232(−), propofol, and midazolam of P6 mice, we next compared the depth of sedation by the righting-reflex latency test. The righting-reflex latency (i.e., length of time needed for the mouse to correct its posture) was evaluated every 5 min following a single injection of anaesthetic. A previous study showed that 9 mg kg^−1^ was within the subanaesthetic range for midazolam in infant mice^25^. Since we did not have accurate information on the subanaesthetic dose of propofol for infant mice, we tested several doses of propofol (20 mg kg^−1^, 40 mg kg^−1^, and 60 mg kg^−1^). The mean latency at each time point was similar for 10 mg kg^−1^ JM-1232(−), 9 mg kg^−1^ midazolam, and 40 mg kg^−1^ propofol (Fig. 1b). There were no significant differences between the three treatment groups in mean latency from 0 to 60 min post-injection (n = 10 or 11 mice for each time point; *p* > 0.05 at all time points, Kruskal–Wallis test). Therefore, we concluded that 10 mg kg^−1^ JM-1232(−), 9 mg kg^−1^ midazolam, and 40 mg kg^−1^ propofol have equivalent sedative effects on P6 mice.

### JM-1232(−) induces a small increase in apoptosis in the brain during development but is less than that induced by other anaesthetics

**Figure 2:**
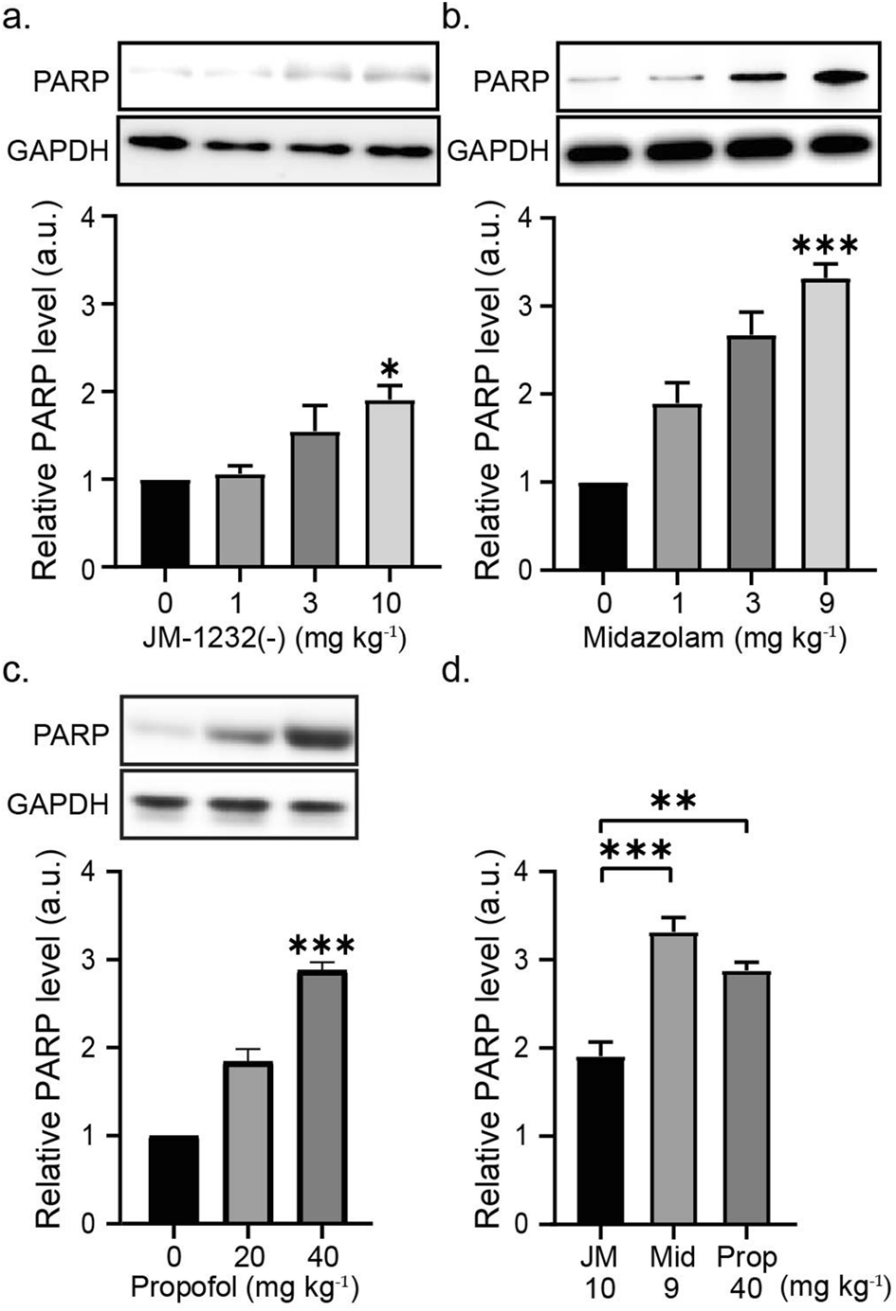
JM-1232(−) induces a small increase in apoptosis in the developing mouse forebrain, but this increase is less than that induced by other anaesthetics. Comparison and quantitation of amount of apoptosis resulting from different doses of JM-1232(−) with comparable doses of midazolam and propofol. Western blot analysis demonstrated that expression levels of cleaved PARP increased in a dose-dependent manner 6 h post-anaesthetic injection in P6 mouse pups. (**a**-**c**) Protein extracts of the forebrain were analysed for cleaved PARP immunoreactivity. GAPDH was used as a loading control. In order to evaluate apoptosis levels, the PARP protein band intensities were normalised to those of the loading control. (**a**) JM-1232(−) (0 mg kg^−1^ [n = 5]; 1 mg kg^−1^ [n = 4]; 3 mg kg^−1^ [n = 4]; 10 mg kg^−1^ [n = 5]). (**b**) midazolam (0 mg kg^−1^ [n = 5]; 1 mg kg^−1^ [n = 5]; 3 mg kg^−1^ [n = 5]; 9 mg kg^−1^[n = 7]). (**c**) Propofol (0 mg kg^−1^ [n = 5]; 20 mg kg^−1^ [n = 5]; 40 mg kg^−1^ [n = 5]). (d) JM-1232(−) induced lower levels of apoptosis when compared with those by propofol or midazolam. Apoptosis levels at the dose of equivalent sedative effect were normalised to apoptosis levels in the corresponding vehicle controls. Comparisons of the means among groups were performed using Kruskal-Wallis test followed by Dunn post hoc test (a) and one-way ANOVA followed by Tukey post hoc test (*p < 0.05, **p < 0.01, ***p < 0.001). Error bars are SEM. Full-length blots are presented in Supplementary Fig. 1.

To investigate neurotoxic effects of JM-1232(−) on the developing mouse brain, we evaluated apoptotic cell death in the forebrain extracts using western blot analysis. Apoptotic cell death was detected by the presence of cleaved poly-(adenosine diphosphate-ribose) polymerase (PARP), a nuclear enzyme involved in apoptosis as described previously^5^. Compared with vehicle controls, administration of 1 mg kg^−1^, 3 mg kg^−1^, or 10 mg kg^−1^ of JM-1232(−) resulted in a small but significant expression of cleaved PARP 6 h after injection (Fig. 2). The amount of cleaved PARP expression was concentration-dependent (n = 4 or 5 mice for each dose; Kruskal–Wallis statistic = 8.33, *p* = 0.025, Kruskal–Wallis test; Fig. 2a).

Next, we evaluated the neurotoxic effects of midazolam in P6 pups. Consistent with the results from these studies^25^, we observed that midazolam (9 mg kg^−1^) increased significantly more cleaved PARP expression 6 h after the injection than the vehicle control mice, and that the amount of cleaved PARP expression was concentration-dependent (n = 5–7 mice for each dose; *F* = 30.3, *p* < 0.0001, one-way ANOVA; Fig. 2b).

We also evaluated the neurotoxic effects of propofol. Previous studies indicated that neonatal administration of propofol induces widespread apoptosis in the brain^7,26,27^. Consistent with the results from these studies, our western blot analyses showed that 6 h after injection propofol significantly increased expression of cleaved PARP more than the vehicle control mice, and the amount of cleaved PARP expression was concentration-dependent (n = 5 mice for each dose; *F* = 94.9, *p* < 0.0001, one-way ANOVA; Fig. 2c). When apoptosis levels at the respective dose of equivalent sedative effect were normalised to each anaesthetic’s corresponding vehicle control, JM-1232(−) produced significantly less apoptosis than that produced by propofol or midazolam (JM-1232(−) [n = 5], midazolam [n = 7], propofol [n = 5]; *F* = 23.5, *p* < 0.0001, one-way ANOVA; Fig. 2d). All vehicle groups (solvents for JM-1232(−), midazolam, or propofol) were statistically indistinguishable in the apoptosis level among each other (Supplementary Fig. S3 online).

### Combined administration of JM-1232(−) and sevoflurane does not potentiate apoptosis compared with sevoflurane administration alone

**Figure 3:**
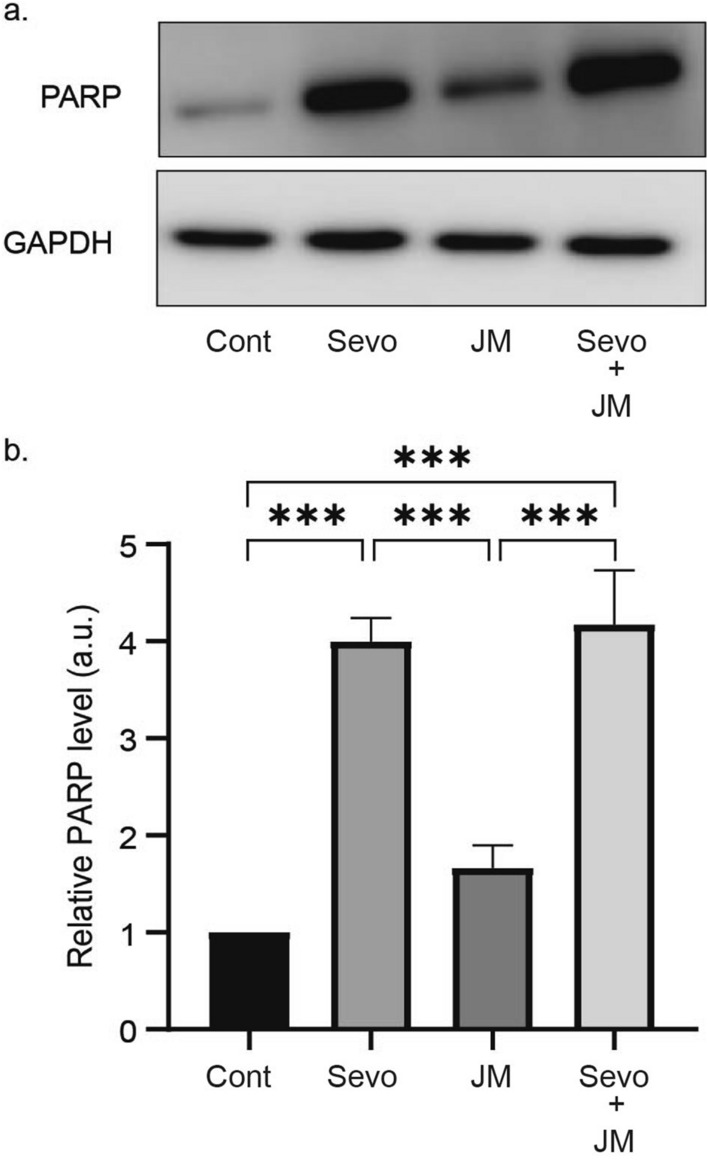
Combined administration of JM-1232(−) and sevoflurane does not potentiate apoptosis in P6 mice compared with sevoflurane administration alone. Western blot analysis showed that the amount of cleaved PARP detected stet for co-administration and single-administration of sevoflurane and/or JM-1232(−). (**a**) Protein extracts of the forebrain of P6 mice were processed and analysed for cleaved PARP immunoreactivity (**b**) and normalised against GAPDH. JM-1232(−) 0 mg kg^−1^ + sevoflurane 0% (n = 6); JM-1232(−) 10 mg kg^−1^ + sevoflurane 0% (n = 6); JM-1232(−) 0 mg kg^−1^ + sevoflurane 2% (n = 6); JM-1232(−) 10 mg kg^−1^ + sevoflurane 2% (n = 6). A comparison of the means among groups was performed using two-way ANOVA followed by Tukey post hoc test (***p < 0.001). Error bars are SEM. Full-length blots are presented in Supplementary Fig. 2.

Combined administration of inhalation and intravenous anaesthetics are used widely in clinical practice. A previous study indicated that sevoflurane in combination with propofol appears to induce more apoptosis than sevoflurane alone in the neonatal mouse brain, although the reason is unclear^28^. Sevoflurane is currently the most widely-used inhalation anaesthetic and is sometimes used in combination with propofol^29^. By contrast, thiopental, another intravenous anaesthetic, did not exacerbate the neurotoxicity of sevoflurane^28^. In the current study, we investigated whether apoptosis induced by sevoflurane is enhanced by JM-1232(−) injection. P6 mice were injected with 10 mg kg^−1^ of JM-1232(−) and administered 2% sevoflurane by inhalation for 6 h, or were given each drug separately. Western blot analysis of forebrain homogenates of the single administration groups showed a slight increase in cleaved PARP level in the JM-1232(−) group but a large significant increase in the sevoflurane group (Fig. 3). The group that received the combination anaesthetics showed about the same level of apoptosis as the sevoflurane group alone. Two-way ANOVA revealed a significant main effect of sevoflurane exposure (n = 6 mice for each group; *F* = 86.0, *p* < 0.0001), but no significant main effect of JM-1232(−) administration (*F* = 2.0, *p* > 0.05). The interaction was not significant (*F* = 0.67, *p* > 0.05). Although 2% sevoflurane exposure significantly increased the expression levels of cleaved PARP more than the vehicle in the control group, the co-administration of JM-1232(−) and sevoflurane did not significantly alter the expression level compared with that of sevoflurane exposure alone (Fig. 3). Thus, JM-1232(−) does not enhance the apoptosis induced by sevoflurane.

### JM-1232(−) has no detectable long-term effects on performance on cognitive function tests

Previous animal studies indicated that neonatal exposure to general anaesthetics induces long-term adverse effects on cognitive functions^1,3^. Thus, we performed behavioural studies to compare any long-lasting effects of JM-1232(−) (10 mg kg^−1^), propofol (40 mg kg^−1^), and midazolam (9 mg kg^−1^) on the developing mouse brain when the mice reached adulthood (11–12 weeks old).

**Figure 4:**
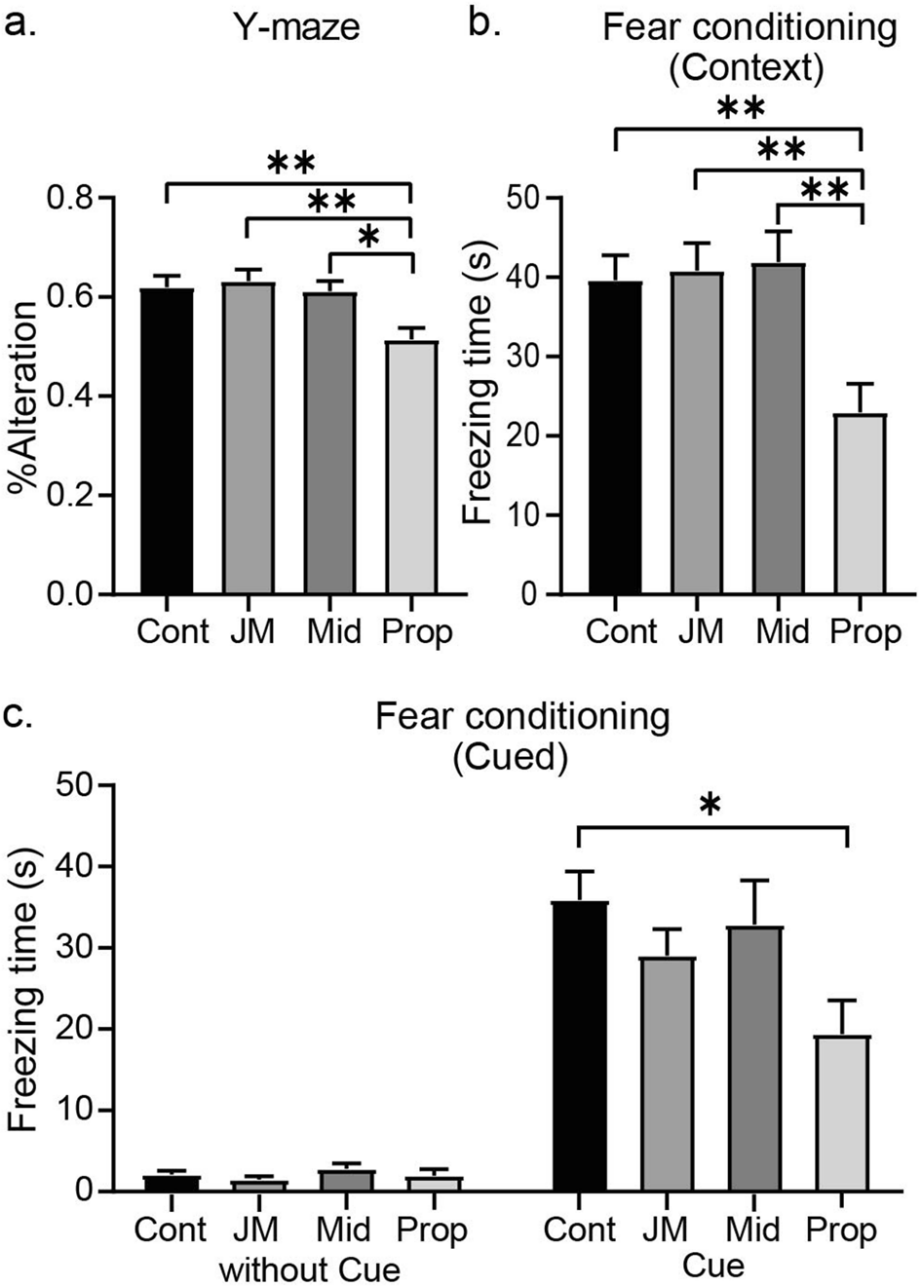
Neonatal administration of JM-1232(−) has no detectable long-term effects on performance on cognitive function tests. Performance of adult mice on behavioural tests of cognitive function after exposure to a single comparable dose of either JM-1232(−), propofol, or midazolam as neonates (P6). (**a**) Mean percentage of correct arm alternations in the Y-maze. Short-term spatial working memory in adulthood was significantly worse in the propofol group (n = 14) compared to that in the control group (n = 14), JM-1232(−) group (n = 14), or midazolam group (n = 13). (**b**) Mean freezing time in contextual fear conditioning. Neonatal exposure of mice to propofol but not JM-1232(−) or midazolam resulted in significantly impaired memory performance in contextual fear conditioning. Freezing response time was measured in the conditioning chamber (contextual fear response) 24 h after conditioning. (**c**) Mean freezing response time in an alternative context with no CS (auditory cue) (Left, basal freezing after conditioning) or with the CS (Right) 48 h post-conditioning. The same set of mice tested in the Y-maze was tested in the fear conditioning tests. Comparisons of the means among groups were performed using one-way ANOVA followed by Tukey post hoc test (*p < 0.05, **p < 0.01). Error bars are SEM.

To assess short-term spatial working memory, mice were subjected to the Y-maze test. Memory performance of mice in the JM-1232(−) group was indistinguishable in adulthood from those in the control group (Fig. 4a). By contrast, mice in the propofol group made fewer correct choices (i.e., lower percentage alternation) compared with the adult mice in the JM-1232(−) group and midazolam group. One-way ANOVA and post hoc tests confirmed these differences (vehicle control [n = 14], JM-1232(−) [n = 14], midazolam [n = 13], propofol [n = 14]; *F* = 5.69, *p* = 0.002; Fig. 4a).

To assess associative memory, they were also tested for contextual/cued fear conditioning. Fear conditioning is a classical pavlovian model to assess associative memory for the pairing of a neutral CS (tone-cue) and the context with an aversive US (electric foot shock)^30^. Once this pairing is acquired, either the context or the tone-cue alone induces the conditioned reaction, i.e. freezing behaviour (non-movement except for respiration). In the contextual test (vehicle control [n = 14], JM-1232(−) [n = 14], midazolam [n = 13], propofol [n = 14]; *F* = 6.60, *p* < 0.001, one-way ANOVA; Fig. 4b), mean freezing time for the propofol group was significantly less (i.e., worse memory) than the other groups. In the cued test (with cue; *F* = 3.07, *p* = 0.036, one-way ANOVA; Fig. 4c), mean freezing time in the propofol group was significantly less than the vehicle control group. These results indicate that neonatal administration of a single dose of JM-1232(−) or midazolam has minimal effects on some memory functions in adulthood, but propofol adversely affects memory function in adulthood.

### Neonatal administration of JM-1232(−) has no detectable effect on sociability in adulthood

Mice are social animals and display clear social interaction behaviours^31^. We previously found that neonatal exposure to sevoflurane leads to social impairment in adulthood^3^. Sociability is a prominent behavioural aspect in autism-related behaviours. We used a rodent sociability test to assess a treated mouse’s preference for another animate mouse (social) versus an inanimate mouse (stuffed toy mouse).

**Figure 5:**
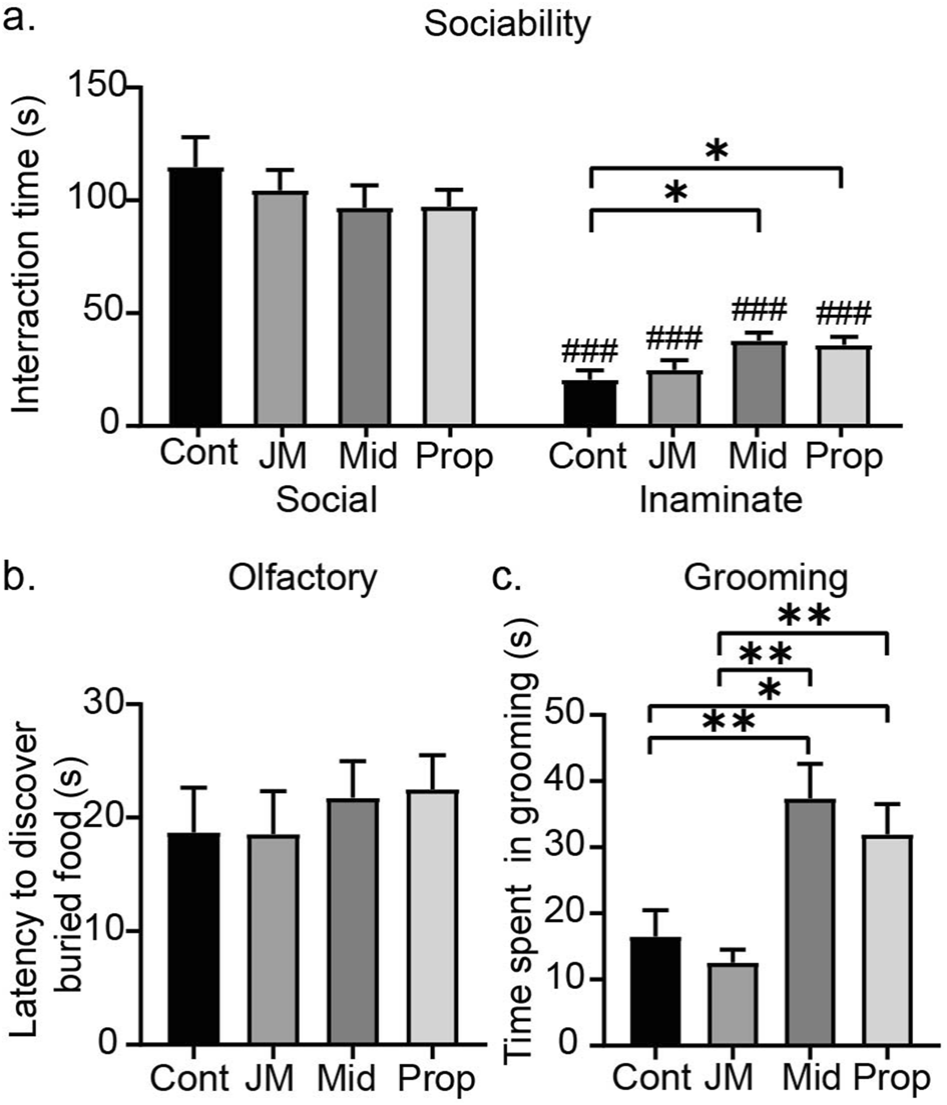
Neonatal administration of JM-1232(−) has no detectable effect on sociability and grooming behaviours in adulthood. Comparison and quantitation of performance on social tests in adulthood after administration of JM-1232(−), midazolam, and propofol in P6 mice. (**a**) Mean interaction time of treated mice when exposed to caged animate mouse (social target) and toy stuffed mouse (inanimate target) in an open field. All groups showed a normal preference for the social target (left) over the inanimate target (right). However, mice receiving midazolam or propofol as neonates showed longer interaction times with the inanimate target than did control mice or mice receiving JM-1232(−) as neonates. Comparisons of the means among groups were performed using one-way ANOVA followed by Tukey post hoc test (*p < 0.05). Comparisons of the means within-group (social vs. inanimate) were performed using Mann–Whitney test (###*p* < 0.001). (**b**) Mean latency to find buried food in the olfactory test. All anaesthetic groups’ latencies were statistically indistinguishable. (**c**) Mean grooming behaviour. Neonatal treatment with midazolam and propofol, but not JM-1232(−), led to longer time spent in adulthood grooming compared to controls. A comparison of the means among groups was performed using Kruskal–Wallis test followed by Dunn post hoc test (*p < 0.05, **p < 0.01). The same set of mice whose performance is presented in Fig. 4 was used in Fig. 5. Error bars are SEM.

Regardless of anaesthetic type administered in P6 mice, in adulthood they spent more time interacting with an animate real mouse than an inanimate fake mouse (*p* < 0.001 for all groups, Mann–Whitney test within group comparison; Fig. 5a). There were no significant differences among groups in time spent interacting with an animate target (vehicle control [n = 14], JM-1232(−) [n = 14], midazolam [n = 13], propofol [n = 14]; *F* = 0.685, *p* > 0.05, one-way ANOVA; Fig. 5a, left). While all mice spent more time interacting with the real mouse (animate), mice treated with the different anaesthetics displayed different behaviours towards the fake mouse (*F* = 4.62, *p* = 0.0062, one-way ANOVA; Fig. 5a, right). The mean times spent interacting with the inanimate target were significantly longer for mice in the midazolam and propofol groups compared to that in the control group. These results indicate that neonatal exposure to midazolam or propofol leads to social impairment in adulthood. In the olfactory test, the anaesthetic groups were indistinguishable from each other and the controls (vehicle control [n = 14], JM-1232(−) [n = 14], midazolam [n = 13], propofol [n = 14]; *F* = 0.339, *p* > 0.05, one-way ANOVA; Fig. 5b). These results suggest that neonatal anaesthetic exposure leads to little or no disruption of olfactory sensation in adulthood, and thus, social impairment is not attributable to impairments of olfactory sensation.

A restricted repetitive and stereotyped pattern of behaviour, expressed in self-grooming in rodents, was affected differently by the anaesthetics tested. This kind of repetitive stereotyped behaviour is another important behavioural aspect in autism^32^. The time spent grooming in adulthood was different among groups (vehicle control [n = 14], JM-1232(−) [n = 14], midazolam [n = 13], propofol [n = 14]; Kruskal–Wallis statistic = 20.76, *p* = 0.0001, Kruskal–Wallis test; Fig. 5c). That for mice in the midazolam and propofol groups was significantly longer compared to that in the control and JM-1232(−) groups. These results indicate that neonatal administration of JM-1232(−) has minimal effects on the social functions and grooming behaviours assessed in these tests, but midazolam and propofol have detectable adverse effects. These results are further supported by the results of the three-chamber social approach task (see Supplementary Fig. S6 online).

### Depression-like behaviours in adulthood are not apparent after neonatal JM-1232(−) administration

**Figure 6:**
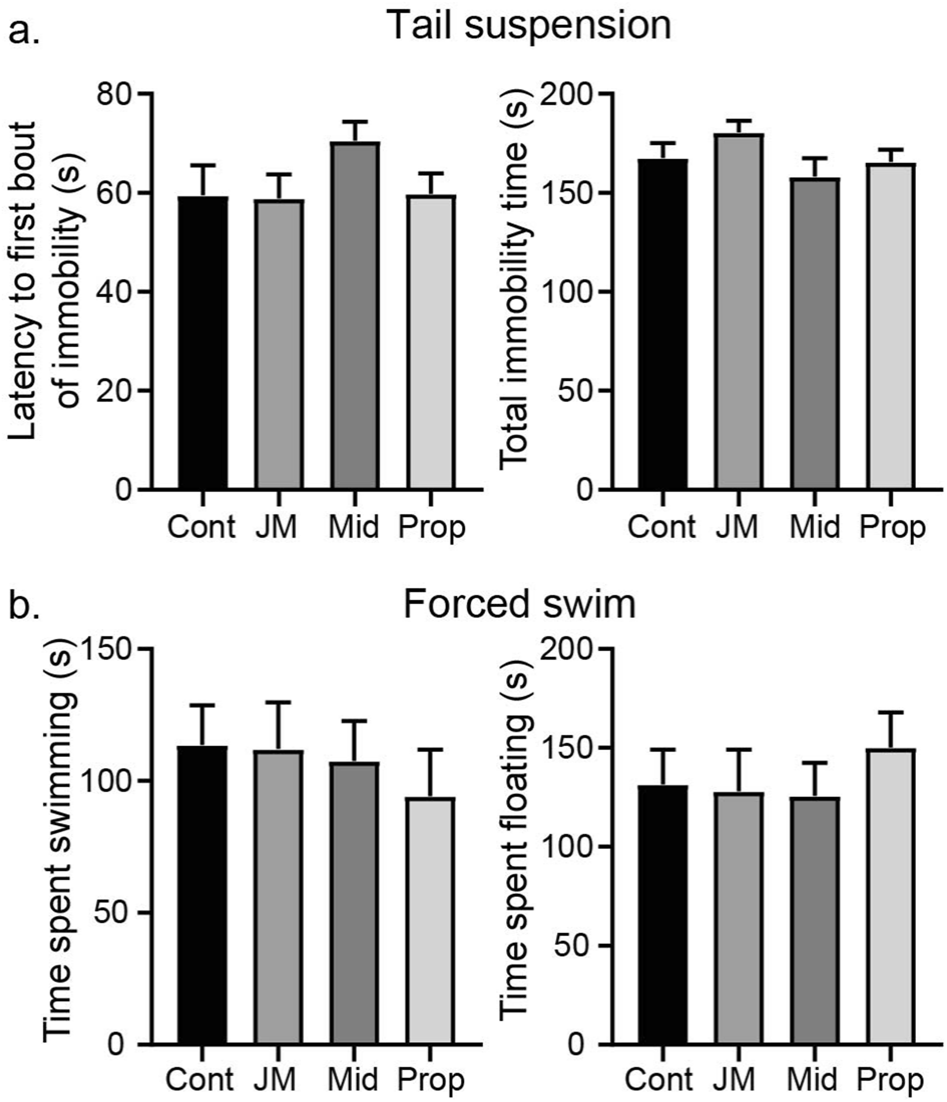
Neonatal administration of JM-1232(−) does not induce depression-like symptoms in adulthood. Comparison and quantitation of performance on ‘depression’ tests in adulthood after administration of JM-1232(−), midazolam, and propofol in P6 mice. (**a**) Mean latencies to immobility (left) and mean total immobile time (right) in tail suspension test. All anaesthetic groups were statistically indistinguishable on both measures among each other and the controls. (**b**) Mean time spent swimming (hopeful) in a pool without an escape platform (left) and mean time spent floating (hopeless) (right) in the forced swim test. Again, all anaesthetic groups were statistically indistinguishable among each other and the controls on both measures. The same set of mice whose performance is presented in Figs. 4 and 5 was used for the depression tests. Error bars are SEM.

To investigate whether neonatal administration of JM-1232(−) affects depression-like symptoms, we tested the treated and control mice on the tail-suspension test, a method for assessing depression-like symptoms^33^. In this test, we measured the latency to the first bout of immobility and the total time spent immobile during a 6-min trial. There were no significant differences among groups in the latency to the first immobility bout (vehicle control [n = 14], JM-1232(−) [n = 14], midazolam [n = 13], propofol [n = 14]; *F* = 1.31, *p* > 0.05, one-way ANOVA; Fig. 6a, left), nor in total time spent being immobile during a 6-min test (*F* = 1.63, *p* > 0.05, one-way ANOVA; Fig. 6a, right).

The same mice were also evaluated on the forced swim test, another method for assessing depression-like symptoms. There were no significant differences among groups in either active swimming time (vehicle control [n = 14], JM-1232(−) [n = 14], midazolam [n = 13], propofol [n = 14]; *F* = 0.296, *p* > 0.05, one-way ANOVA; Fig. 6b, left) or in floating (immobility) time (*F* = 0.377, *p* > 0.05, one-way ANOVA; Fig. 6b, right). These results indicate that neonatal administration of JM-1232(−), midazolam, and propofol have minimal effects on depression-like behaviours assessed in adulthood.

## Discussion

There is growing concern about the use of general anaesthetics in obstetric and paediatric practice^34^. Although extrapolating from animal models to humans can be fraught with difficulties, results from animals suggest that repeated or lengthy use of general anaesthetic during surgery in children younger than 3 years may affect brain development, which can be manifested later in adulthood as cognitive problems. The mechanisms underlying the neurotoxicity of general anaesthetics in the developing brain are not yet fully understood, and thus, effective alternative anaesthetic is still lacking. Assessing potential new anaesthetics in animal models is the first step in determining whether a particular anaesthetic can be safely used in clinical setting.

Although neurodevelopmental age equivalencies between mice and humans cannot be specified with precision, our decision to use 6-d-old pups for the present study was based on the assumption that this neurodevelopmental age in the mouse is equivalent to the critical period of the human age^35^. The critical period is a distinct time-window in the neonatal stage when animals display elevated sensitivity to certain environmental stimuli, and particular experiences can have long-lasting and profound effects on behaviours. The critical period of neurobehavioral development is a time of learning opportunity but also of vulnerability for interruption: interruption during the critical period can cause irreversible and permanent problems in the brain. It is well known that the closure of one eye (monocular deprivation) of a kitten during the critical period results in loss of visual acuity in the deprived eye despite no physical damage to the eye itself^36^. Studies about anaesthetic-induced toxicity in brain development indicate that anaesthetic-induced apoptosis is the greatest if exposure occurs at P6–P7^1,3,37^, with little or no increase in apoptosis at P14 in rodents^37^. Furthermore, neonatal exposure of pups to anaesthetics at P6-P7 could cause behavioural impairments later in adulthood^1^, including social deficits similar to those seen in autistic spectrum disorder^3^. Thus, there may be a critical period of vulnerability for the exposure to anaesthetics with the peak at P6–P7 and the critical period would be closed before P14 in rodents.In the current study, we found that a single injection of JM-1232(−) at subanaesthetic dose resulted in a small increase in apoptotic cell death in the developing mouse brain. However, the level of apoptosis resulting from JM-1232(−) was significantly lower than that resulting from dose-equivalent sedative effects of propofol or midazolam. Immunohistochemical analysis confirmed that the number of cells with activated (cleaved) caspase-3^+^ (AC3^+^) significantly increased in mice treated with anaesthetics at P6 compared to vehicle controls (Supplementary Fig. S4 online). However, the number of AC3^+^ cells in the JM-1232(−) group was significantly lower than that of the propofol or midazolam groups (Supplementary Fig. S4 online). Distribution patterns of degenerating neurons in all three types of anaesthetics are similar to the pups with sevoflurane exposure in our previous studies^3,38^; the increased apoptosis is most robust in the retrosplenial cortex, subiculum, neocortex, and thalamus in the brains of pups with anaesthetic exposure (Supplementary Fig. S4 online).

Most importantly, we did not detect any behavioural deficits when mice reached adulthood after neonatal JM-1232(−) injection, in contrast to the other widely-used general anaesthetics. However, a causal link between apoptosis appearing immediately after anaesthesia and behavioural deficits manifested later in life remains unknown.

Our results indicate that toxicity of JM-1232(−), midazolam, and propofol are different, although the underlying mechanism is unknown. General anaesthetics modulate specific ligand-gated ion channels, principally GABA_A_ receptors and/or *N*-methyl-d-aspartate (NMDA) receptors, the latter being a subtype of glutamate receptors. Several studies have demonstrated that NMDA receptor-mediated and GABA_A_ receptor-mediated responses both have adverse effects on the developing brain^4,7,10,39^. A previous study suggested that apoptosis is synergistically potentiated when both NMDA and GABA_A_ receptors are simultaneously affected in the developing brain^4^. It is well known that exposure of the foetus to ethanol during critical periods of development induces foetal alcohol syndrome. Ethanol acts through both NMDA and GABA_A_ receptors. In this context, although propofol acts mainly by binding to GABA_A_ receptors, it also acts, to a lesser extent, by binding to NMDA receptors^20^. JM-1232(−), on the other hand, binds to GABA_A_ receptors, and it barely inhibits glutamine receptors^40^. Thus, the different effects of propofol and JM-1232(−) on the developing brain might result from different effects of the two drugs on the NMDA receptor.

Propofol is sometimes co-administered with sevoflurane being one of the common inhalation anaesthetics^29^. Sevoflurane binds to GABA_A_ receptors similar to many intravenous anaesthetics^41,42^. A previous study reported that 3% sevoflurane in combination with 10 mg kg^−1^ propofol induces a more robust apoptosis than sevoflurane alone in the neonatal mouse brain^28^. In contrast, our results demonstrated that co-administration of 2% sevoflurane with JM-1232(−) (10 mg kg^−1^) did not significantly increase the level of apoptosis compared with 2% sevoflurane alone. These differences also suggest that the mechanisms of toxicity of JM-1232(−) and propofol likely differ.

Previous studies showed that exposure of the immature brain to benzodiazepine can result in cognitive alterations lasting long after the cessation of benzodiazepine exposure, although results of these studies are inconsistent^43–45^. This inconsistency probably relates to differences in the schedules of drug administration, different ages among those tested, different drugs, and differences in tests. In consistent with our results, Mikulecka et al. reported that neonatal exposure to rats (from P7 until P11) with therapeutically relevant doses of clonazepam, a classic benzodiazepine, lead to disturbances of cognitive-like behaviour^46^, and social deficits later in adulthood^47^. Although both midazolam and JM-1232(−) act by binding to the benzodiazepine site of GABA_A_ receptors^16^, only mice administered midazolam as neonates exhibited social impairments and enhanced grooming behaviour during adulthood in the current study, even though both anaesthetics were given at sedative-equivalent dosages. While the mechanisms underlying the neurotoxicity of midazolam and JM-1232(−) are not well understood, it may be noteworthy that the molecular structures of the two are significantly different (i.e., benzodiazepine structure of midazolam vs. nonbenzodiazepine structure of JM-1232(−)).

In the current study, mice received only a single injection of JM-1232(−) at P6. Previous reports suggest that repeated administration of general anaesthetics may cause more severe neurotoxicity compared to a onetime administration^27^. Thus, our results might have been different if we had exposed mice to multiple episodes of JM-1232(−).

Because of the animal-model caveat, the relevance of our findings in mice to human neonates is unknown. Thus, it is too early to say definitively whether JM-1232(−) has the same mild effect in humans. However, it is reasonable to propose the idea that JM-1232(−), or its derivatives, could be more favourable as an obstetric and paediatric anaesthetic, since its neurotoxic effects are minimal on the developing mammalian brain at least one instance of an animal model. With better knowledge in the future, we would be able to anaesthetize pregnant mothers and infants safely. Until then, we would be to select anaesthetics that are the least harmful.

## Methods

### Animals

All experiments were conducted according to the institutional ethical guidelines for animal experiments of the National Defense Medical College (Tokorozawa, Japan). The experimental protocol was approved by the Committee for Animal Research at the National Defense Medical College (approval number: 17074). This study was carried out in compliance with the ARRIVE guidelines (http://www.nc3rs.org.uk/page.asp?id=1357).

Inbred, C57BL/6 male mice were used in this study (CLEA Japan, Inc., Tokyo, Japan). Age of the mice ranged from P6, when anaesthesia was administered, to 11–12 weeks of age, when behavioural tests were administered. The mice were housed under standard laboratory conditions, with a 12-h light/dark cycle and a room temperature maintained at 23 ± 1 °C. The mice were given access to water and food ad libitum.

In total, n = 314 mice were used in the study. However, one pup in the midazolam group was died unexpectedly prior to the behavioural tests, preventing analysis, and another pup was died during the anaesthesia with propofol (60 mg/kg). The mortality during the anaesthesia was 11.1% (one mouse died in 9 mice treated anaesthesia) for the propofol (60 mg/kg) group and 0% for other groups.

### Preparation of anaesthetic solutions

The 5.0 mg ml^−1^ JM-1232(−) solution was prepared as follows: 141 µl of 0.1 mol L^−1^ HCl was added to 5 mg of JM-1232(−) powder (Maruishi Pharmceutical Co. Ltd., Osaka, Japan), then 0.009 g of NaCl and sterile water were added to final volume of 1 ml. The solution was adjusted to pH 5.0 using 0.1 mol L^−1^ NaOH. The 5.0 mg ml^−1^ of JM-1232(−) solution was diluted to the indicated concentrations with sterile saline. Assuming the body weight of the subject mouse to be 5 g, we injected each mouse intraperitoneally with 10 μl of 0.5 mg ml^−1^, 1.5 mg ml^−1^, 2.5 mg ml^−1^, or 5.0 mg ml^−1^ solutions, which correspond to 1.0 mg kg^−1^, 3.0 mg kg^−1^, 5.0 mg kg^−1^, or 10 mg kg^−1^, respectively. Control mice received an injection of only vehicle solution (i.e., 0 mg kg^−1^ dose of anaesthetic).

Propofol (FUJIFILM Wako Pure Chemical, Osaka, Japan) containing same amount of soybean oil was diluted to the indicated concentrations with Intralipos Injection 10% (Otsuka Pharmaceutical, Tokyo, Japan). Intralipos Injection was used as a soybean-oil-enriched lipid emulsion. Assuming the body weight of the subject mouse to be 5 g, we injected each mouse intraperitoneally with 10 μl of 10 mg ml^−1^, 20 mg ml^−1^, or 30 mg ml^−1^ solutions, which correspond to 20 mg kg^−1^, 40 mg kg^−1^, or 60 mg kg^−1^, respectively.

Midazolam (5 mg ml^−1^; Sandoz, Tokyo, Japan) was dissolved with sterile saline and diluted to the indicated concentrations with sterile saline. Assuming the body weight of the subject mouse to be 5 g, we injected each individual mouse with 10 μl of 0.5 mg ml^−1^,1.5 mg ml^−1^, or 4.5 mg ml^−1^ solutions, which corresponded to 1.0 mg kg^−1^, 3.0 mg kg^−1^, or 9 mg kg^−1^, respectively.

Exact body weight of pups was measured just prior to anaesthesia treatment and calculate proper volume of injection. Then, pups were intraperitoneally injected with the calculated volume of anaesthetics using a 10-μL Hamilton syringe (Hamilton Bonaduz AG, Bonaduz, Switzerland) with a 30G 1/2″ needle. The body temperature of pups was kept at 37 ± 1 °C during the anaesthesia using the circulating thermal water system (Gaymar MUL-T- Blanket Hyper/Hypothermia Blanket https://www.meenamedical.com/catalog/pc/Gaymar-MUL-T-Blanket-Hyper-Hypothermia-Blanket-p335.htm) under pups.

Sevoflurane (2,2,2-trifluoro-1-[trifluoromethyl]ethyl fluoromethyl ether) inhalation anaesthesia was carried out as described previously^5^. Briefly, pups were placed in a humid chamber and sevoflurane (2%) was administered in 30% oxygen as the carrier gas for 6 h. The total gas flow was 2 L min^−1^. Concentrations of sevoflurane and oxygen were monitored with a Datex-Ohmeda S/5 monitor (GE Healthcare, Chicago, IL USA). For simultaneous administration of sevoflurane and JM-1232(−), pups were placed in a chamber with sevoflurane immediately after JM-1232(−) injection. The body temperature of pups was kept at 37 ± 1 °C during the anaesthesia as described above.

### Western blot analysis

Western blot analysis was performed as described previously^5^. Immediately after the end of anaesthesia treatment for 6 h, pups were subjected to decapitation. Then, whole forebrain was removed and extracting protein from tissues lysates was subjected to SDS-PAGE. We used at least four pups per group for western blot analysis. The proteins were transferred onto polyvinylidene fluoride membranes (Immobilon-P, Millipore, Bedford, MA USA), and the blots were immunoreacted with primary antibodies. The primary antibodies used were anti-PARP (#9544, rabbit polyclonal; Cell Signaling Technology, Beverly, MA USA) and anti-GAPDH (#3683, rabbit monoclonal horseradish peroxidase [HRP] conjugate, Cell Signaling Technology). The secondary antibodies used were HRP-linked anti-rabbit IgG (#7074, goat polyclonal, Cell Signaling Technology) and HRP-linked anti-mouse IgG (#7076, horse polyclonal, Cell Signaling Technology). The protein bands were visualised with a chemiluminescence detection system (ImmunoStar LD; FUJIFILM Wako Pure Chemical, Osaka, Japan). Blot images were captured on a luminescent image analyser (Amersham Imager 600, GE Healthcare, Tokyo, Japan). Full length western blot images are shown in Supplementary Figs. S1-3 online.

### Loss of righting reflex test

Dose-dependency of JM-1232(−)’s sedative effects was evaluated by the LORR test^48^. Twenty minutes after the mouse received a single injection of anaesthetic, the animal was placed on its back gently and scored as LORR if it failed to completely right itself within 30 s. One trial was given.

### Righting-reflex latency test

Depth of sedation after an injection of anaesthetic was evaluated with the righting-reflex latency test. The animal was placed on its back carefully as in the LORR test and the latency to correct its posture was measured. Cut-off time for a failure was set at 300 s. Measurements were started immediately after the injection, and then taken every 5 min.

### Behavioural studies

Behavioural studies were performed as described previously^49^. Mice were randomly assigned to the vehicle (solvent for JM-1232(-)); JM-1232(−) (10 mg kg^−1^); midazolam (9 mg kg^−1^); or propofol (40 mg kg^−1^) group. To control for litter variability, we divided the litters evenly into groups of equal size. Because it was difficult to prepare litter with more than four male pups, we used only solvent for JM-1232(-) as vehicle control in the behavioural studies. However, all vehicle groups (solvents for JM-1232(−), midazolam, or propofol) were statistically indistinguishable among each other on all measures (Supplementary Fig. S5 online).

Mice were administered a single intraperitoneal injection at P6. Immediately after anaesthetic treatment for 6 h as described above, pups were immediately returned to their real dams (pups were separated from their dams for 6 h). And then, pups were weaned at 4 weeks of age. Controls were handled the same way as treated animals. Care was taken to handle the mice gently during treatment and observations to minimize stress.

Behavioural tests were performed in the mice at 11–12 weeks of age. The same set of mice was used for all behavioural tests (vehicle [n = 14]; JM-1232(−) [n = 14]; midazolam [n = 13]; propofol [n = 14]). The order of the behavioural tests was as follows: Y-maze test, sociability test, grooming test, olfactory test, tail-suspension test, forced swim test, and fear-conditioning test. Individual mice underwent only one test per day. Each experimental observation was performed by the same experimenter, who was blinded to the treatment conditions. The apparatus was cleaned after each trial.

### Y-maze test

The Y-maze test evaluates spatial working memory^50^ and was administered as described previously^51^. Briefly, each mouse was placed in the centre of the Y-maze, after which it was allowed to freely explore the maze for 8 min. The sequence of arm entries and total number of arms entered were recorded. An arm entry was counted when all four limbs of the mouse were completely on an arm. The percentage of alternations was the number of triads containing entries into all three arms divided by the maximum possible number of alternations (total number of arm entries minus 2) × 100. Fewer correct choices can indicate poorer spatial working memory.

### Fear-conditioning test

The fear-conditioning test assess associative memory^30^ and was performed as previously described^49^. Briefly, the conditioning trial for contextual and cued fear conditioning consisted of a 5-min exploration period followed by three conditioned stimulus-unconditioned stimulus pairings (CS-US), each separated by a 1 min intertrial interval (US: 1 mA foot shock intensity, 1 s duration; CS: 80 db white noise, 20 s duration; US was delivered during the last seconds of CS presentation). A context test was administered to a trained mouse 24 h after conditioning in the conditioning chamber in the absence of white noise (chamber exposure, 5 min). The same set of mice underwent cued test 48 h after conditioning. In this test, the cue (80 db white noise, 3 min duration) was presented in an alternative, novel context having distinct visual and tactile cues. The mouse’s rate of freezing response (total number of min with absence of movement ÷ 5 min) was used to measure its fear memory.

### Sociability test

The preference for interaction with an animate (caged naïve adult mouse) versus inanimate (caged dummy mouse) targets (sociability) was examined in an open-field chamber (50 × 50 × 40 cm, made of white acrylic), as described previously^52^. Animate or inanimate targets were put into cylindrical cages, allowing olfactory, but minimal tactile, interactions. The cylinder cages were 10 cm in height, with a bottom diameter of 9 cm and vertical bars spaced 7 mm apart. Total time sniffing directed at the cage was measured for 10 min under 70 lx lighting conditions.

### Olfactory test

The olfactory test was conducted as described previously^49^. Briefly, on the first day of the test, mice were habituated to the flavour of a novel food (blueberry cheese). After 48 h of food deprivation, the mouse was placed in a clean cage, and the time elapsed for the mouse to find the buried piece of blueberry cheese was measured. The blueberry cheese was buried under 2 cm of test cage bedding.

### Tail-suspension test

The tail-suspension test, which evaluates depression-like symptoms, was administered as described previously^35^. Briefly, a mouse was suspended from the edge of a desk by attaching its tail to the desk with adhesive tape. The adhesive tape was placed approximately 5–10 mm from the tip of the tail. The suspended animal was 600 mm away from the floor. The total duration of immobility (i.e., lack of movement with the head pointed downward) and the latency to the first episode of immobility were measured during a 6 min test period.

### Forced-swim test

The forced-swim test, another assay that measures depression-like symptoms in rodents, was administered as described previously^35^. Briefly, a mouse was placed in a cylinder (25 cm diameter, 46 cm depth) filled two-thirds with water (25 ± 1 °C) for 6 min. The mouse could not escape from the cylinder, and its feet could not touch the bottom of the cylinder. Naïve mice typically swim in the water to search for an escape route from the water. This test measures the time spent swimming versus the time spent floating. Swimming behaviour was defined as active horizontal movement through the water that exceeds that necessary to merely maintain the head above the water. It is a proxy measure to assess ‘hopefulness’. Floating behaviour was defined as lack of movement beyond what is necessary to maintain balance and to keep the nose above water; it was measured as a proxy variable of ‘hopelessness’. After each trial, the mouse was lightly towel-dried and introduced back into its home-cage. The water in the cylinder was changed between each animal.

### Statistical analysis

Statistical analysis was performed using GraphPad Prism 8 (GraphPad Software Inc, La Jolla, CA USA). Sample size was defined according to previous studies^38,49,53,54^. To obtain ED_50_ value, we used a variable slope model (nonlinear fit [agonist] *versus* normalized response–variable slope, GraphPad Prism 8). Statistical comparisons between the means of each treatment group were performed using t-test, one-way analysis of variance (ANOVA), or two-way analysis of variance (ANOVA) followed by a Tukey post hoc test, when the assumption of normality was satisfied. When the assumption of normality was not satisfied, comparisons were made using Mann–Whitney test or Kruskal–Wallis test, followed by a Dunn post hoc test. Data are presented as means ± standard error of the mean (SEM). The ED_50_ values were presented with 95% CI.

## Supplementary Information

Supplementary Information.

## Data Availability

Data are available from the corresponding author upon a reasonable request and with permission of Dr. Yasushi Satoh.
