# Supplementary material for: Neonatal administration of a subanaesthetic dose of JM-1232(−) in mice results in no behavioural deficits in adulthood
Source: Sci Rep. 2021 Jun 18;11:12874. doi: 10.1038/s41598-021-92344-3 (PMC8213711; doi:10.1038/s41598-021-92344-3)

**Supplementary Information**

# Neonatal administration of a subanaesthetic dose of JM-1232(−) in mice results in no behavioural deficits in adulthood

Koji Iwanaga[1], Yasushi Satoh[1,2,3], Ryosuke Akai[1], Toshiaki Ishizuka[3], Tomiei Kazama[4], Takehiko Ikeda[1].

[1]Department of Anesthesiology, National Defense Medical College, Tokorozawa, Saitama, Japan.

[2]Department of Biochemistry, National Defense Medical College, Tokorozawa, Saitama, Japan.

[3]Department of Pharmacology, National Defense Medical College, Tokorozawa, Saitama, Japan.

[4]Department of Anesthesia, Toyooka-Daiichi Hospital, Iruma, Saitama, Japan.

Correspondence Author: Yasushi Satoh, Department of Pharmaceutcal Science, National Defense Medical College, 3-2 Namiki, Tokorozawa 359-8513, Japan.

E-mail: wndlt3@gmail.com

Phone: +81-4-2995-1452

Fax: +81-4-2992-1215

## Methods

### Three-room chambered social approach task

Sociability and social preference for novelty were evaluated in the three-room chambered social approach task[1, 2]. These tests were performed in the mice at 12 weeks of age. Conditions for anaesthetic treatment and experimental grouping were same as other tests described in the main text.

Each chamber was 20 cm long × 40.5 cm wide × 30 cm high. Dividing walls were made from clear Plexiglas, with small openings allowing access into each chamber. The cylindrical cages used were the same as those used in the sociability test in the open field (see "Sociability test" in the Method of main text). The apparatus was cleaned after each trial. In the test, mice were initially allowed to explore the chambers for 10 min. The subject mouse was initially allowed to explore the chambers for habituation for habituation. Each of the two sides contained an empty cage. After the habituation period, a caged male mouse, which had no prior contact with the subject mice, was placed in one of the side chambers (stranger 1). The subject mouse was placed in the middle chamber, and then the mouse was allowed to interact with an empty cage in one room versus a caged social target in another room for 10 min

(sociability test). At the end of the ten-minute sociability test, each mouse was further tested in a third ten-minute session to quantitate preference to spend time with a new stranger (preference for social novelty test). A new unfamiliar stranger mouse was placed in an identical cage in the chamber (stranger 2) that had been empty during the previous ten-minute session. The test mouse had a choice between the first, already-investigated, now-familiar mouse (stranger 1) and the novel unfamiliar mouse (stranger 2). The time spent in each chamber was measured.

Stranger mice were adult male C57BL/6J, and were housed in cages separate from and distant to the cages housing the subject mice, to avoid visual, auditory, and olfactory contact. Strangers had no previous physical contact with the subjects, and were kept in a separate location from the subjects on the day of testing.

**Immunohistochemistry**

Immunohistochemical analysis using an antibody against activated caspase-3, biomarker of apoptotic cell death, was performed as previously described[3]. Briefly, animals were fixed with 4% paraformaldehyde in phosphate-buffered saline (PBS, 10 mM, pH 7.4) under anaesthesia. Brain was removed and post-fixed in 4% paraformaldehyde in PBS for 1 h. Tissues were embedded in paraffin using standard

procedures. Paraffin sections (5 µm thick) were de-paraffinized and immersed in an antigen unmasking solution (Vector H3300; Vector Laboratories, Burlingame, CA) for antigen retrieval. The sections were then heated in an autoclave (121°C) for 5 min. Next, the sections were incubated with a blocking reagent (Dako, Glostrup, Denmark) for 30 min to reduce nonspecific background staining. The sections were incubated with primary antibody against activated caspase-3 (#9661, rabbit polyclonal, Cell Signaling Technology, Danvers, MA) overnight in a humidified chamber at 4°C. Immunoreactivity (IR) was detected using peroxidase-conjugated secondary antibody (Dako EnVision+ system; Dako) and 3,3'-diaminobenzidine (DAB) tetrahydrochloride (Vector Laboratories). Sections were coverslipped and examined using a self-contained bright-field digital microscope (COOLSCOPE II, Nikon) with 20× magnification at a resolution of 2560×1920. Immunostained phospho-AC3-IR cells of the cortex layer II, retrosplenial cortex (RSC), subiculum, and thalamus were counted in sections from the brain. Only cells with clearly visible IR nuclei were counted. Two adjacent sections (5 µm per section) were selected per individual (n = 5 mice for each group) and the IR numbers were averaged across the two sections.

**REFERENCES**


1. Satoh, Y. *et al.* ERK2 contributes to the control of social behaviors in mice. *J Neurosci* **31**, 11953-11967 (2011).
2. Moy, S.S. *et al.* Mouse behavioral tasks relevant to autism: phenotypes of 10 inbred strains. *Behav Brain Res* **176**, 4-20 (2007).
3. Yufune, S. *et al.* Suppression of ERK phosphorylation through oxidative stress is involved in the mechanism underlying sevoflurane-induced toxicity in the developing brain. *Scientific reports* **6**, 21859 (2016).

## FIGURE LEGENDS

**Supplementary Figure S1. Full length western blot images to accompany Figure 2.**

**(a-c)** To investigate neurotoxic effects of JM-1232(−) (**a**), midazolam (**b**), and propofol (**c**) on the developing mouse brain, we evaluated apoptotic cell death in the forebrain extracts using western blot analysis. Apoptotic cell death was detected by the presence of cleaved poly-(adenosine diphosphate-ribose) polymerase (PARP). Left and right panels show the expression of cleaved-PARP and GAPDH, respectively. Samples were run on a 10% polyacrylamide gel. For molecular weight marker (M, in kDa), we used Full-Range Amersham Rainbow Marker (RPN800E; GE Healthcare Bio-Sciences AB, Uppsala, Sweden). Firstly, we detected PARP, and then, GAPDH as a loading control on the same membrane. We did not use stripping procedure.

**Supplementary Figure S2. Full length western blot images to accompany Figure 3.**

Western blot analysis showing that the amount of cleaved PARP detected differed for co-administration and single-administration of sevoflurane and/or JM-1232(−). Blot condition was same as mentioned in Supplementary Figure S1.

**Supplementary Figure S3. The cleaved PARP level in mice treated vehicles for JM-1232(−), midazolam, or propofol.**

Western blot analysis showed that there were no significant differences in brain apoptosis among pups administered vehicles for JM-1232(−), midazolam, or propofol at P6. (**a**) Protein extracts of the forebrain were analysed for cleaved PARP immunoreactivity. GAPDH was used as a loading control. Quantified band intensities were normalized to those of the loading control. Further, the band intensities were normalized to those of the vehicle for JM-1232(−) (n = 6 mice for each group). A comparison of the means among groups was performed using Kruskal-Wallis test (Kruskal-Wallis statistic = 4.71, $p$ = 0.0913). Data are represented as means ± SEM. JMV: vehicle for JM-1232(−), MV: vehicle for midazolam, PV: vehicle for propofol. (**b**) Full length western blot images to accompany the figure in (a). Blot condition was same as mentioned in Supplementary Figure S1.

**Supplementary Figure S4. Immunoreactivity for cleaved caspase-3 in the brain regions from mice administered vehicle, JM-1232(−), midazolam, or propofol.**

Numbers of black dots labelled by immunohistochemical staining for cleaved caspase-3 were increased in the regions of brain from the pups with anaesthetic exposure; the

increased apoptosis was most robust in the retrosplenial cortex (RSC), subiculum, cortex layer II, and thalamus. (**a**) Representative images of the brain regions from mice treated with control (vehicle for JM-1232(−)), JM-1232(−) (10 mg $kg^{-1}$), midazolam (9 mg $kg^{-1}$), and propofol (40 mg $kg^{-1}$). Scale bars: 50 µm. (**b**) Numbers of black dots for cleaved caspase-3. Comparisons of the means among groups were performed using one-way ANOVA (Cortex layer II: $F = 21.3$, $p < 0.0001$, RSC: $F = 30.3$, $p < 0.0001$, Subiculum: $F = 13.0$, $p = 0.0001$, Thalamus: $F = 17.7$, $p < 0.0001$) followed by Tukey *post hoc* test (*$p < 0.05$, **$p < 0.01$, ***$p < 0.001$). Data are represented as means ± SEM.

**Supplementary Figure S5. Vehicles for JM-1232(−), midazolam, or propofol administered at P6 have minimal impact for behaviours later in adulthood.** Mice administered vehicles for JM-1232(−), midazolam, or propofol at P6 were statistically indistinguishable in behavioural outcomes among each other. Behavioural tests were performed as described in Figures 4-6. Comparisons of the means among groups (n = 11 mice for each group) were performed using one-way ANOVA (Y-maze test; $F = 0.141$, $p > 0.05$, fear conditioning test (context); $F = 0.264$, $p > 0.05$, fear conditioning test (cued, without cue); $F = 2.07$, $p > 0.05$, fear conditioning test (cued,

with cue); $F = 0.160$, $p > 0.05$, sociability test (social); $F = 0.068$, $p > 0.05$, sociability test (inanimate); $F = 0.409$, $p > 0.05$, olfactory test; $F = 0.844$, $p > 0.05$, tail suspension test (first bout); $F = 0.267$, $p > 0.05$, tail suspension test (total); $F = 1.03$, $p > 0.05$, forced swim test (swimming); $F = 0.410$, $p > 0.05$, forced swim test (floating); $F = 0.126$, $p > 0.05$)) and Kruskal-Wallis test (grooming test, Kruskal-Wallis statistic = 1.79, $p > 0.05$). In the sociability test, comparisons of the means within-group (social vs. inanimate) were performed using Mann-Whitney test (###$p < 0.001$). Data are represented as means ± SEM.

**Supplementary Figure S6. Mice administered midazolam or propofol but not vehicle or JM-1232(−) at P6 show altered preference for social novelty in the three-room chambered social approach task later in adulthood.**

(**a**) In the sociability test in the three-room chambered social approach task, time spent in the side with stranger 1 was significantly different from that in the empty cage side in all groups (n = 12 mice for each group). (**b**) In the preference for social novelty test, mice administered vehicle or JM-1232(−) showed a normal preference for social novelty (stranger 2). By contrast, mice administered midazolam showed no preference between the stranger 1 and 2. Mice administered propofol spent significantly less time

in the stranger 2 side than in the stranger 1 side. The same set of mice was used as in (a). **(c)** In the sociability test, time spent in the side with stranger 1 was significantly different from that in the empty cage side in mice administered vehicles for JM-1232(−), midazolam, or propofol (n = 12 mice for each group). **(d)** In the preference for social novelty test, all groups showed a normal preference for social novelty (stranger 2). The same set of mice was used as in (c). Comparisons of the means within-group were performed using Student's *t* test (*$p < 0.05$, **$p < 0.01$, ***$p < 0.001$). Data are represented as means ± SEM.

Supplementary Figure S1

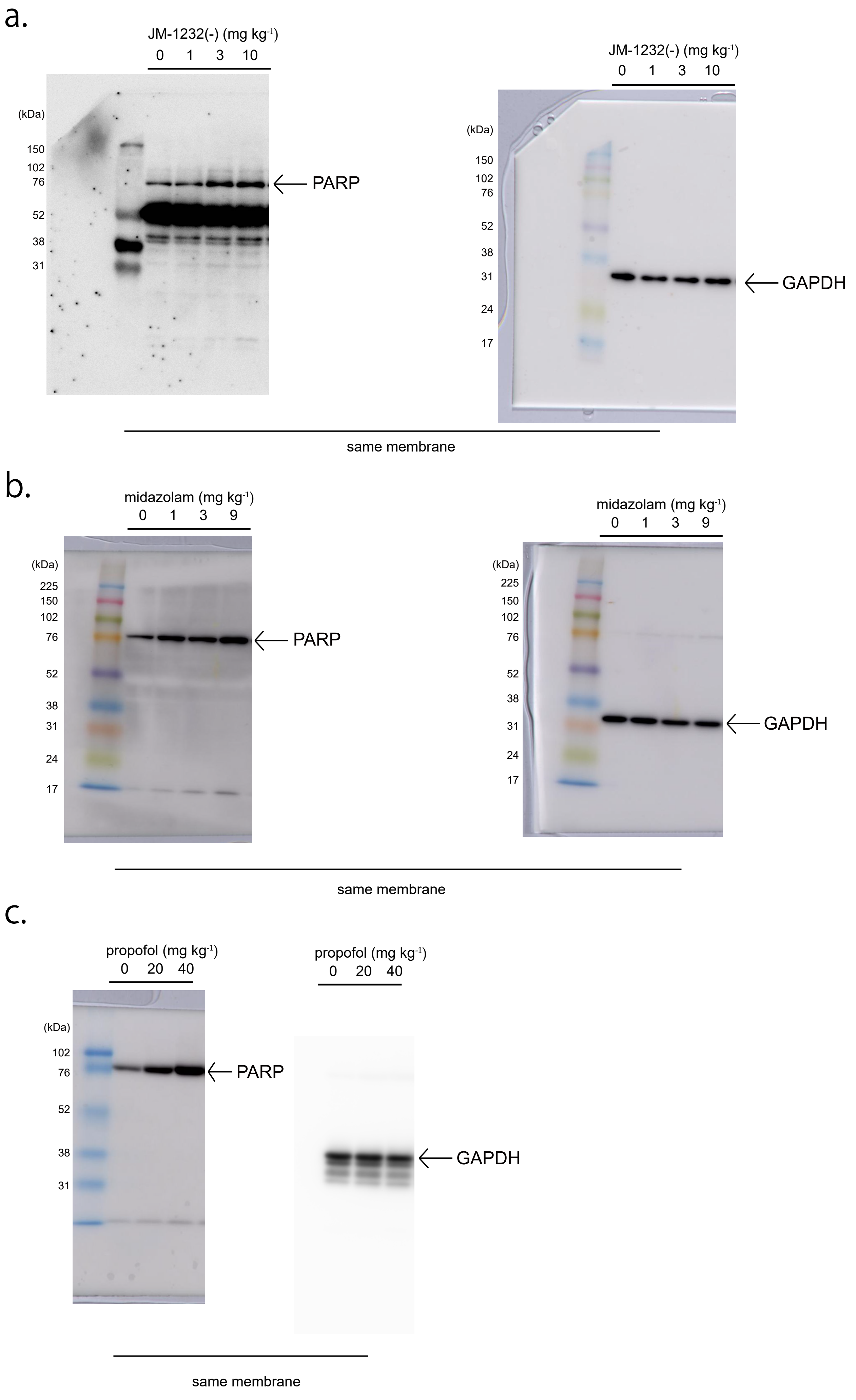

Supplementary Figure S2

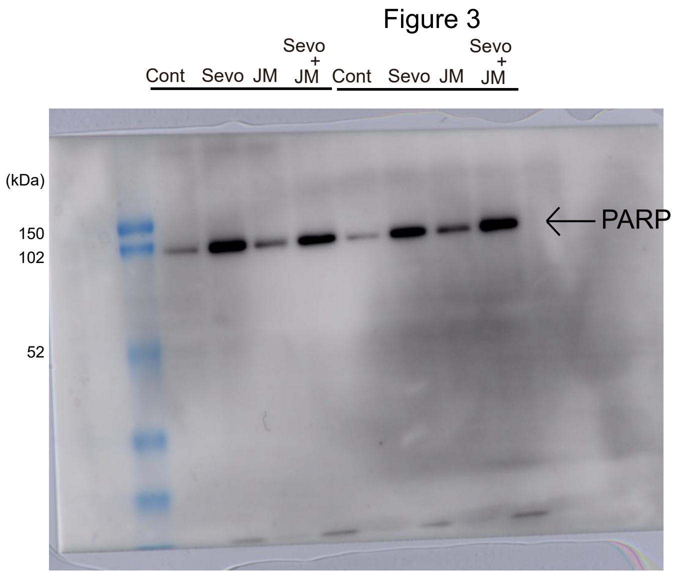


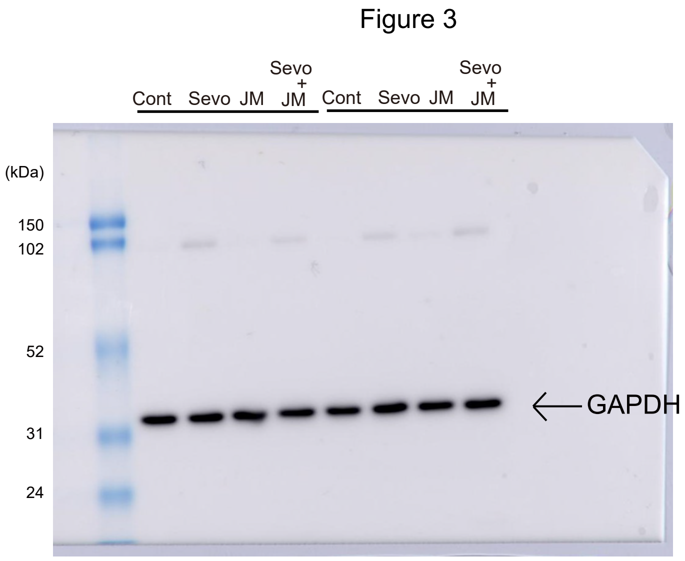


same membrane

Supplementary Figure S3

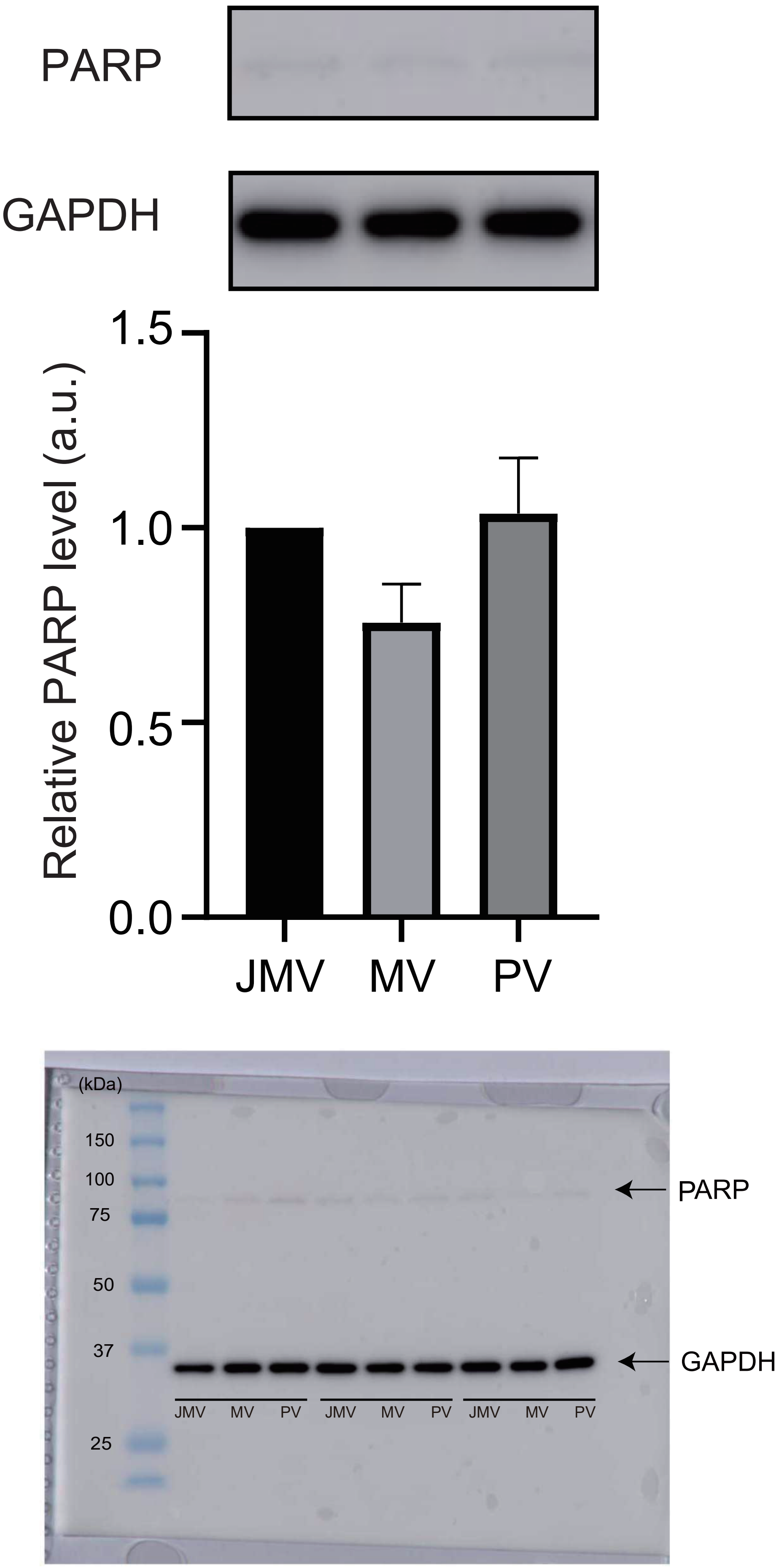

Supplementary Figure S4

a.

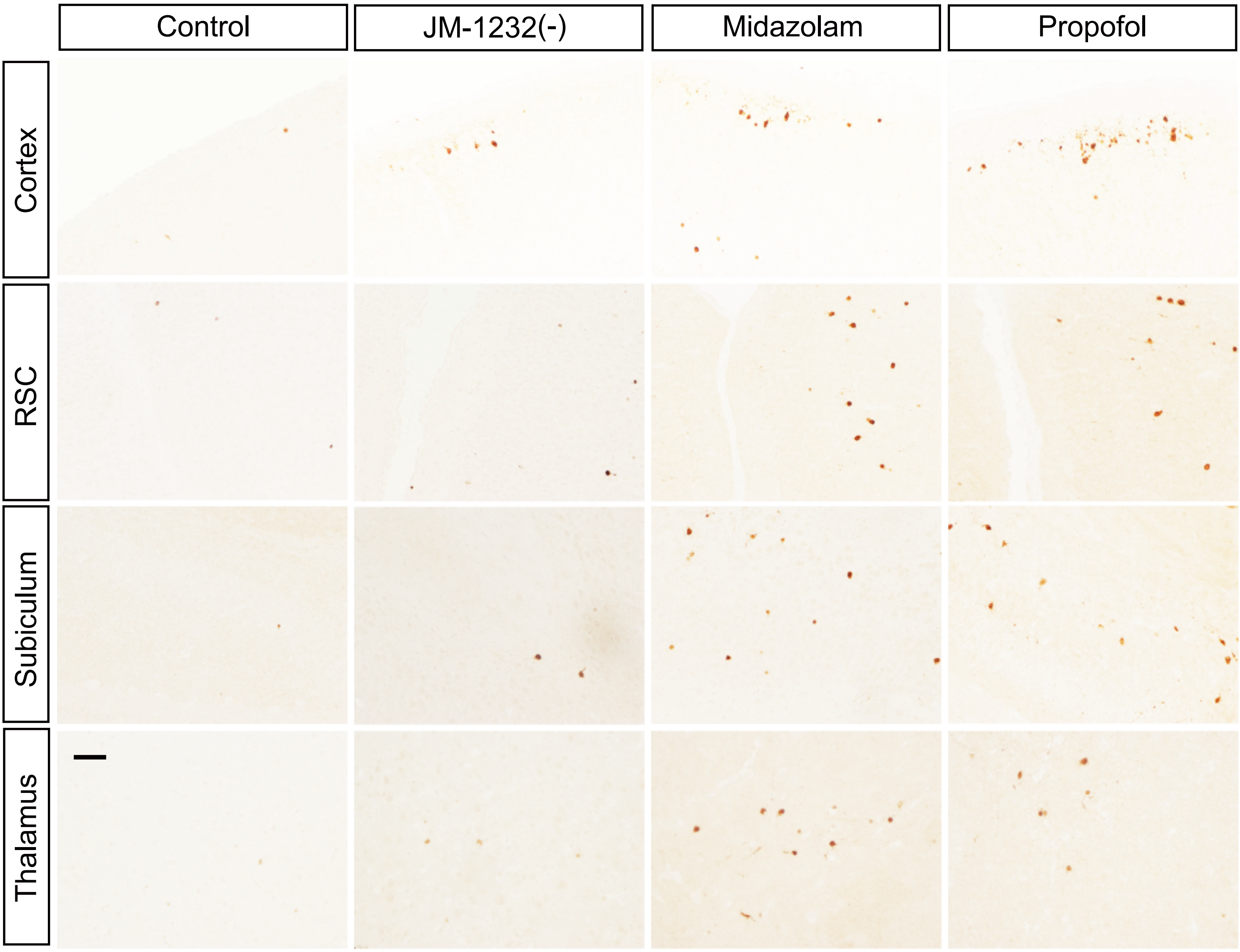


b.

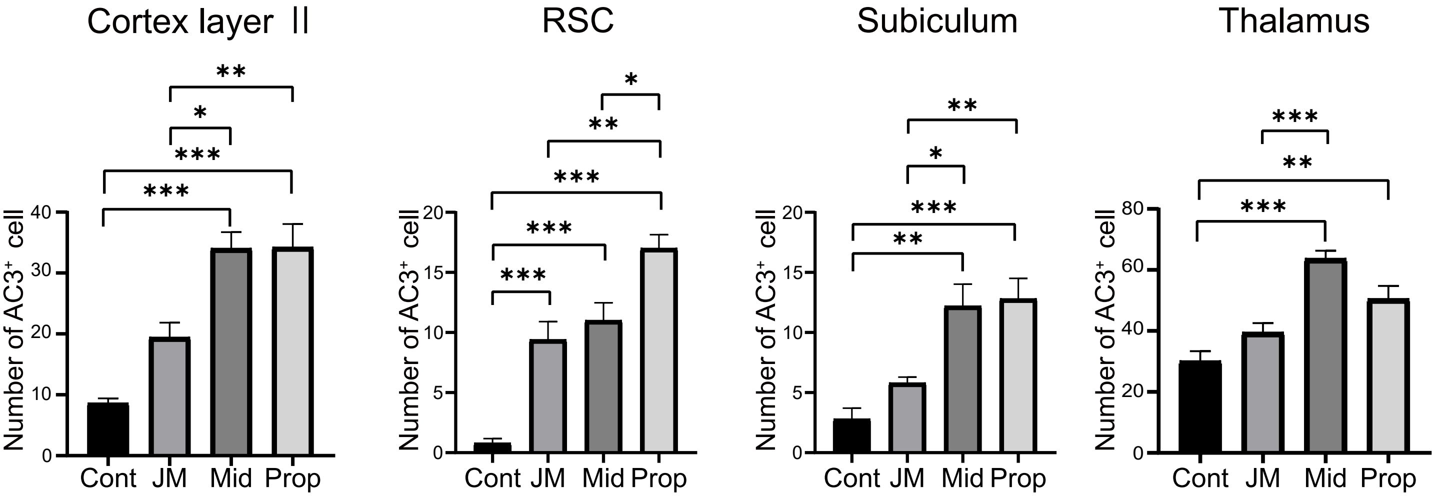

Supplementary Figure S5

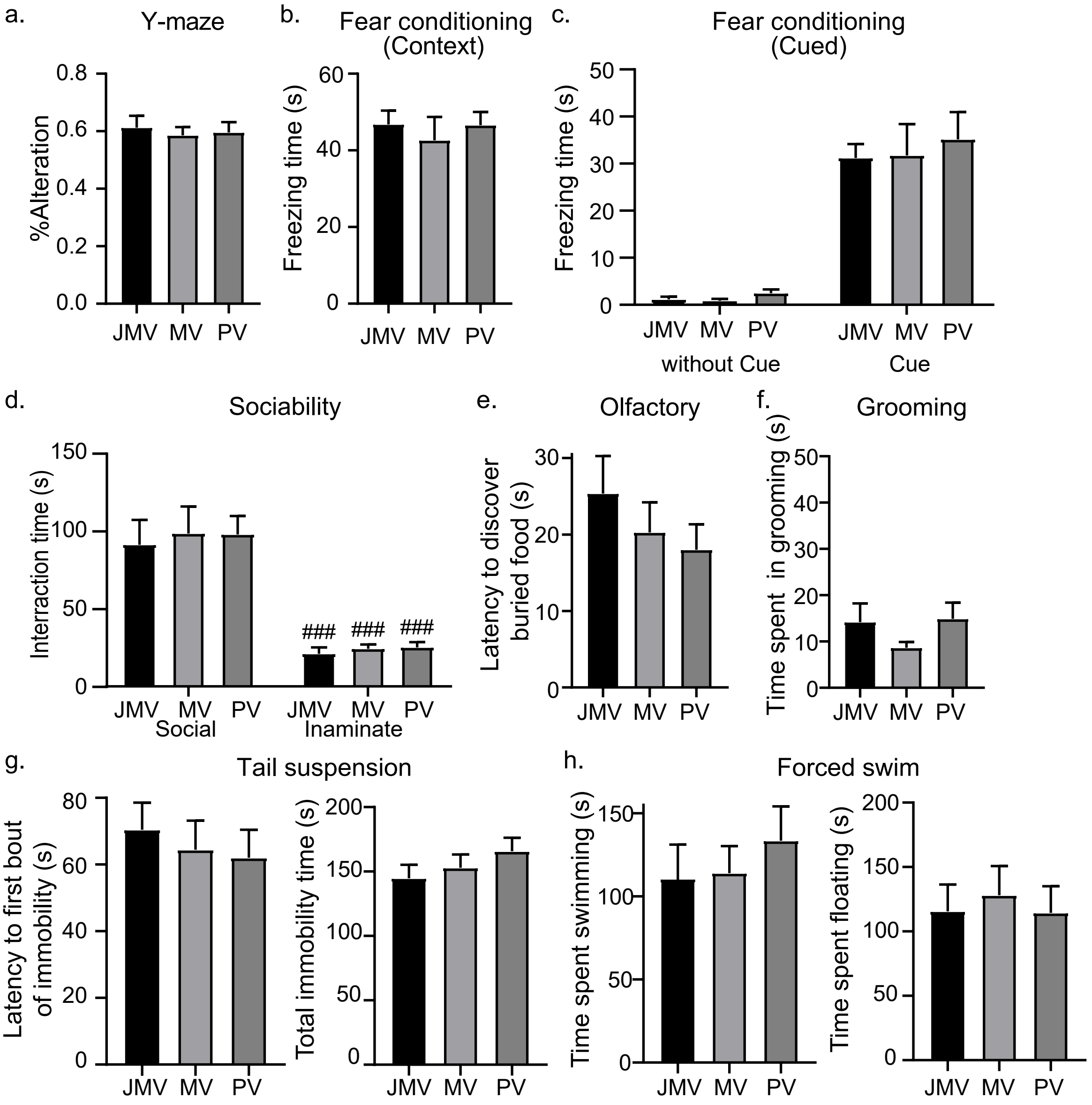

Supplementary Figure S6

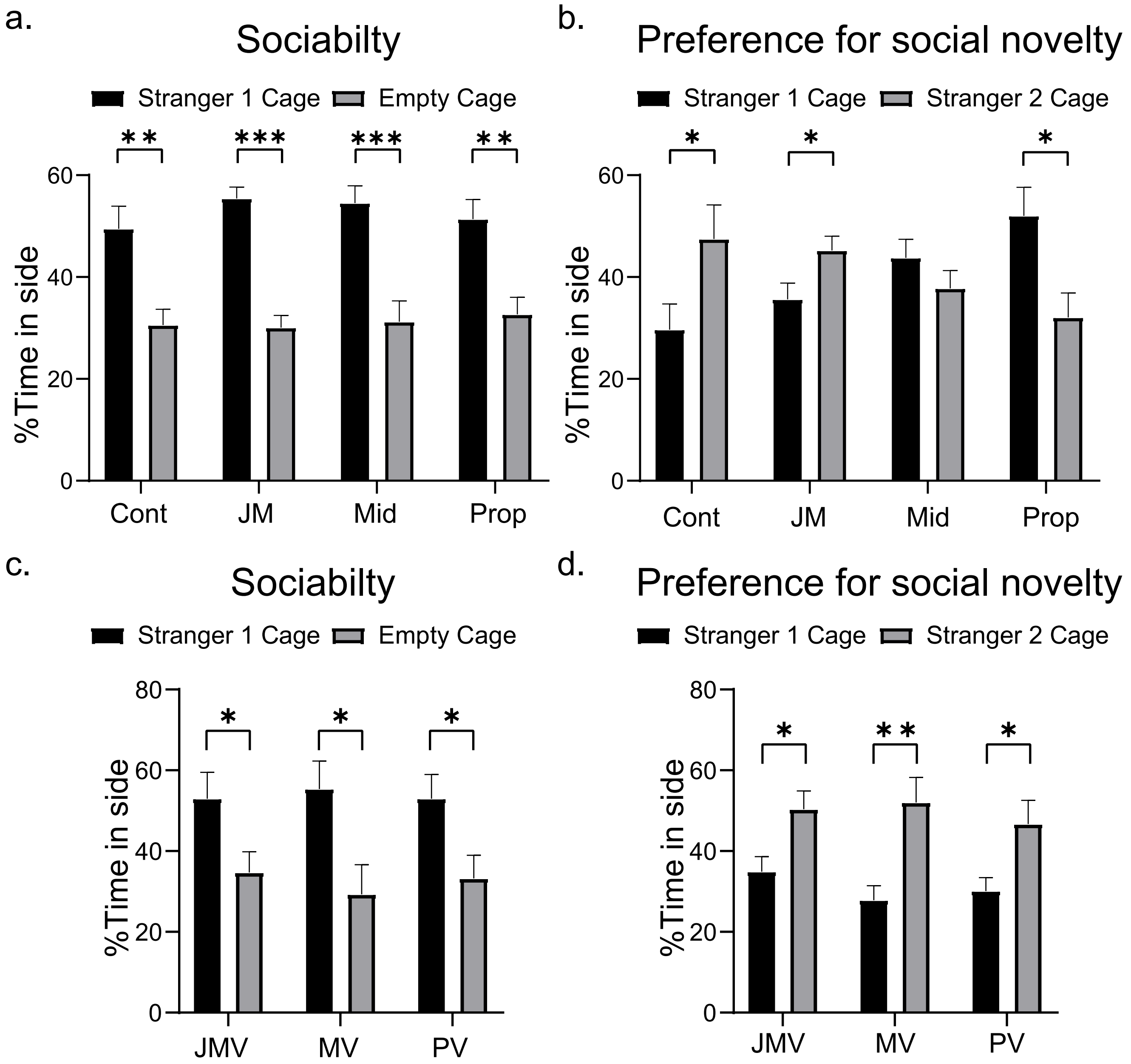

Supplement: Supplementary file 1 — Supplementary Information. [file 41598_2021_92344_MOESM1_ESM.pdf]
